# A practical approach to better identify *NTRK 1–3* fusion-positive mesenchymal neoplasms by pan-Trk immunohistochemistry

**DOI:** 10.1007/s00428-025-04102-9

**Published:** 2025-04-15

**Authors:** Martina Haberecker, Pauline Kuerten, Viola Katharina Vetter, Francesca Malega, Holger Moch, Chantal Pauli

**Affiliations:** 1 Department of Pathology and Molecular Pathology, University Hospital Zurich, Rämistrasse 100, 8091 Zurich, Switzerland; 2 Medical Faculty, University Zurich, Zurich, Switzerland

**Keywords:** NTRK, Immunohistochemistry, Mesenchymal tumors, Antibody clone

## Abstract

Pan-Trk immunohistochemistry has become an affordable screening tool for tumors harboring *NTRK1 - 3*-rearrangements. However, false positive staining has been addressed especially in tumors with mesenchymal origin. As a positive staining triggers reflex testing, a better understanding about pan-Trk immunohistochemistry in these tumors has become necessary. In this work, we extensively studied pan-Trk IHC in a large cohort of mesenchymal neoplasms using two antibody clones: EPR17341 (RTU Assay, Roche/Ventana) and A7H6R (Cell Signaling Technologies). Whole slide sections of 809 individual cases, including 35 subtypes of mesenchymal neoplasms, were analyzed by two different pan-Trk antibodies. Any positivity above background in > 1% of tumor cells was classified as positive. Positive stained cases were molecularly analyzed. The specificity of clone EPR17341 was 78% and showed 21.9% false positive staining (177/809). Forty-five percent (80/177) of the false positive stained cases harbored a non-*NTRK*-gene fusion. When comparing the two antibodies in mesenchymal neoplasms, clone A7H6R showed 80% less false positive stains compared to clone EPR17341. Additionally, three tumors harboring a *NTRK1*-fusion were newly identified (0.4%) and reclassified in our cohort. Our work showed a high false positive rate in mesenchymal neoplasms using clone EPR17341. Clone A7H6R demonstrated a higher specificity and therefore could be considered in clinical practice for screening mesenchymal tumors for *NTRK1 - 3*-rearrangements, eventually leading to less unnecessary reflex testing.

**Supplementary Information:**

The online version contains supplementary material available at 10.1007/s00428-025-04102-9.

## Introduction

The discovery and characterization of oncogenic *NTRK*-fusions led to the development of tumor agnostic targeted therapeutic agents that successfully inhibit Trk fusion proteins [1, 2]. TRK protein expression can be detected using immunohistochemistry, and the European Society of Medical Oncology (ESMO) recommends IHC as a screening tool, particularly in situations where a *NTRK1 - 3-*rearrangement is expected, or sequencing platforms are not available [3]. The available pan-Trk antibodies bind to antigens on the C-terminal domain, which all three TRK proteins (since TrkA, TrkB, and TrkC) have in common. This method relies on the fact that most normal and tumor cells express only low levels of Trk, while *NTRK1 - 3* gene fusion-positive cancer cells typically have elevated Trk protein levels. Variable rates of sensitivity (82–100%) and specificity (81–96%) have been reported in solid tumors [4–6]. False positive staining can occur in tumors lacking *NTRK1 - 3-*rearrangements and has been reported in a meta-analysis as high as 18% [4]. Certain tumor types have a particularly low specificity for pan-Trk IHC, such as breast carcinomas, salivary gland or *BCOR* rearranged tumors [5, 7, 8]. Additionally, TrkA, TrkB, and TrkC play an important role in neural development, consequently, neoplasms showing neuronal or muscle differentiation may often show corresponding wild-type Trk expression [9, 10]. Positivity for pan-Trk IHC always leads to reflex testing for a *NTRK1 - 3*-related gene fusion and can cause unnecessary costs in the context of a false positive stain. In the other hand, pan-Trk IHC is a suitable method for confirming the pathogenicity of fusions, especially when detected with a DNA-based assay. Soft tissue and bone tumors are generally underrepresented in solid tumor studies, due to the rarity and the fact that sarcomas are less likely sequenced in a clinical routine setting. The rarity is problematic due to a tremendous heterogeneity of these tumors. Here, we analyzed 809 whole sections of mesenchymal soft tissue and bone tumors, accounting for 35 different subtypes, by pan-Trk IHC. In cases with positive staining, we additionally performed molecular testing to better understand the sensitivity of pan-Trk immunohistochemistry assays in mesenchymal soft tissue and bone tumors.

## Material and methods

### Sample selection

Eight hundred nine patients diagnosed with a mesenchymal neoplasm in the Department of Pathology and Molecular Pathology, University Hospital Zurich (USZ), Switzerland, between 1997–2023, were included in this study. Archival FFPE from excisions and resection specimens were selected and the diagnosis was confirmed by two expert pathologists (C.P., M.H.). Tumor types included in this study were: adipocytic sarcomas (*n* = 83), undifferentiated pleomorphic sarcomas (UPS) (*n* = 78), leiomyosarcomas (*n* = 62), synovial sarcomas (*n* = 64), malignant peripheral nerve sheath tumors (MPNST) (*n* = 35), solitary fibrous tumors (SFT) (*n* = 33), Ewing sarcomas (*n* = 31), gastrointestinal stroma tumors (GIST) (*n* = 36), angiosarcomas (*n* = 26), myxofibrosarcomas (*n* = 22), rare sarcomas of uncertain differentiation (*n* = 22), rare fusion-positive sarcomas (*n* = 22), fibroblastic and myofibroblastic tumors (*n* = 18), skin sarcomas (*n* = 19), osteosarcomas (*n* = 174), chondrogenic sarcomas (*n* = 16), endometrial stroma sarcomas (*n* = 11), low-grad sarcomas NOS (*n* = 5), benign tumors (*n* = 12), and schwannomas (*n* = 37). Three neoplasms, diagnosed prior to 2013, were re-classified due to a newly identified pathogenic *NTRK1*-rearrangement (*TPR::NTRK1* (2x), *LMNA::NTRK1*) (Table 1 and Supplementary 1).

**Table 1:** Mesenchymal tumors with positive pan-Trk Immunohistochemistry

Entity	EPR17341 Positive/total *N*		A7H6R Positive/total *N*	
Adipocytic sarcomas	23/82	(14%)	0/25	–
UPS	19/78	(24%)	3/31	(10%)
Leiomyosarcoma, NOS	6/63	(1%)	0/16	–
Synovial sarcoma	17/64	(26%)	0/64	–
MPNST	8/35	(23%)	1/17	(6%)
SFT	7/33	(21%)	3/33	(9%)
Ewing sarcoma	12/31	(39%)	2/22	(9%)
Gastrointestinal stromal tumor	16/36	(44%)	7/26	(27%)
Angiosarcoma	0/26	–	0/12	–
Myxofibrosarcoma	0/22	–	0	–
Rare sarcomas of uncertain differentiation	9/22	(41%)	1/22	(5%)
Epitheloid sarcoma	2/9	(22%)	0/9	–
Extraskeletal myxoid chondrosarcoma	3/6	(50%)	1/6	(17%)
Intimal sarcoma	4/7	(57%)	0/7	–
Rare fusion positive sarcomas	9/22	(41%)	1/17	(9%)
Alveolar soft part sarcoma	0/2	–	0	–
DSRCT	2/2	(100%)	0/2	–
CIC-rearranged sarcoma	0/3	–	0	–
Sarcoma with BCOR genetic alterations	4/7	(57%)	0/7	–
Clear cell sarcoma of soft tissue	3/8	(37%)	1/8	(13%)
Fibroblastic and myofibroblastic tumor	1/18	(6%)	0/18	–
Skin sarcomas	6/19	(32%)	2/19	(11%)
Dermatofibrosarcoma protuberans	6/14	(43%)	2/14	(14%)
Kaposi and pleomorphic dermal sarcoma	0/5	–	0/5	–
Osteosarcoma	5/174	–	2/5	(40%)
Chondrogenic sarcomas	3/16	(19%)	0/3	–
Endometrial stroma sarcoma	4/12	(33%)	0/4	–
Low-grade sarcoma NOS**	1/5	(20%)	0/1	–
Schwannoma	31/37	(85%)	12/31	(39%)
Other benign tumor*	1/12	(8%)	0/12	–
NTRK fusion positive tumor	3/3	(100%)	3/3	(100%)
Total	**180/809**	(22%)	37/381	(7%)

*UPS* undifferentiated pleomorphic sarcoma, *MPNST* malignant peripheral nerve sheath tumor, *SFT* solitary fibrous tumor, *IMT* inflammatory myofibroblastic tumor

^*^Other benign tumor: osteochondroma (1), osteoblastoma (3), desmoid fibromatosis (2), vascular malformation (4), myxoma (1), and nodular fasciitis

^**^Not further classifiable: low-grade myofibroblastic tumor (3), low-grade leiomyomatous tumor (1), and low-grade epitheloid tumor (1)

The presented study was conducted following regional/cantonal and institutional guidelines, in compliance with the Helsinki Declaration and after approval by our cantonal ethical review board Zurich (BASEC- 2021–00417).

### Immunohistochemistry

**Fig. 1:**
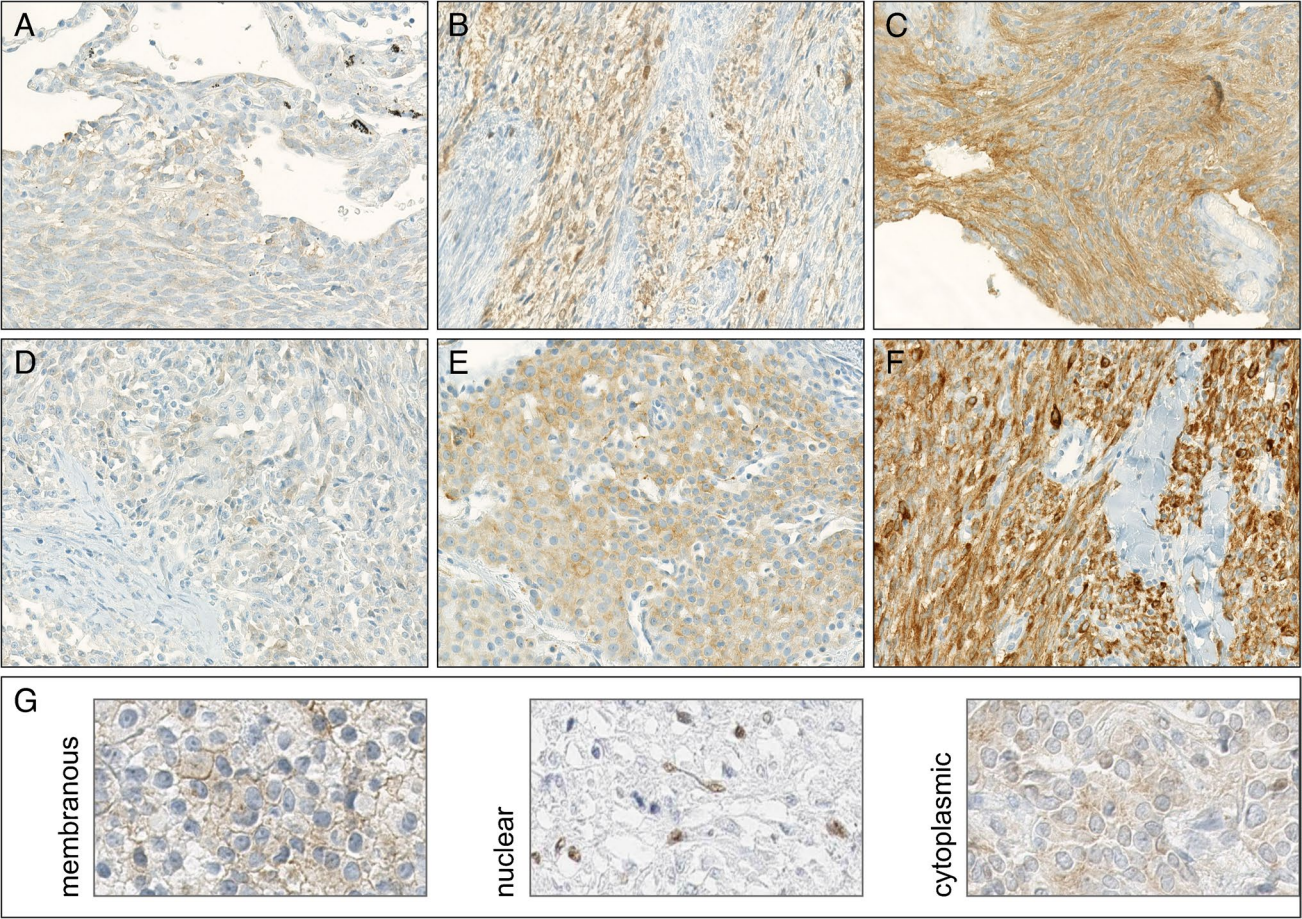
Scoring: semi-quantitative assessment of staining intensity and pan-Trk immunohistochemistry (EPR17341): negative (0, not shown); **A, D** staining above background, weak (score 1); **B, E** moderate (score 2); **C, F** strong (score 3). Magnification 200x. **G** False positivity by compartment: membranous (score 1), nuclear (score 1), and cytoplasmic (score 2). Magnification 400x

Pan-Trk immunohistochemical stains were performed on full sections using VENTANA pan-Trk (EPR17341) ready to use (RTU) Assay by Roche Diagnostics on a Ventana platform (Tucson, AZ) according to the manufacturer’s instructions. For pan-Trk clone A7H6R (Cell Signaling Technologies), epitope retrieval was performed on a Leica Bond platform (Chicago, IL) using H2 (EDTA buffer with pH 9.0) for 60 min on the stainer. The sections were incubated with the monoclonal antibody to pan-Trk (A7H6R, 1:100 dilution) for 30 min, and detection was carried out using Bond Polymer Refine DAB IHC detection kit. Normal appendix vermiformis and cortical brain tissues was used as positive controls. Each IHC slide was read by at least two individual pathologists (M. H., P. K., F. M., V. V., and C. P.). If no agreement between tow pathologists was found, the case was read by a third pathologist. Pathologists were blinded to each other’s scoring results and to the molecular data. Positive staining was defined as staining above background in ≥ 1% of the tumor cells according to published literature [10]. Staining intensity was semi-quantitatively quoted as negative (0), weak (1 +), moderate (2 +), or strong (3 +) (Fig. 1). Additionally, positive compartments (cytoplasmic, membranous, and nuclear) were distinguished. All cases with any positivity using pan-Trk (EPR17341), other fusion positive entities, and randomly picked cases where additionally stained with A7H6R (*n* = 381). Both antibody clones demonstrated the same positive expression level on positive control tissue (appendix vermiformis and cortical brain tissue).

### Molecular testing

Molecular testing was performed on FFPE material using commercially available molecular tumor profiling assays, used and validated for routine diagnostics: custom-made Archer Sarcoma Fusion Plex Assays (RNA) detecting 132 fusions, FoundationOne®CDx (DNA), FoundationOne®Heme (DNA and RNA), Sanger sequencing for entity specific fusion detection (*SSX::SYT*, *FUS::DDIT3*, *EWSR1::FLI1*), and *KIT* or *PDGFRA* alteration (Supplementary 2). DNA and RNA extraction, library preparation, sequencing, and data analysis were done according to clinical protocols following the routine work up in the Department of Pathology and Molecular Pathology, USZ, Switzerland, and as previously published [6]. In total, 415 tumors (51%) were molecularly characterized. This includes score 2 and 3 positive tumors (*n* = 60, EPR17341) with the exception of some schwannomas (35/37) and one MPNST due to technical issue (degraded nucleic acids). Furthermore, 72 score 1 positive tumors (*n* = 72, EPR17341) were analyzed.

Fluorescence in situ hybridization (FISH) was performed using the ZytoLight SPEC *NTRK1* Dual Color Break Apart Probe (PL123) from Zytovision in combination with the ZytoLight FISH-Tissue Implementation Kit (Prod. No. Z- 2028–5/− 20) on formalin-fixed, paraffin-embedded (FFPE) tumor tissue in cases harboring a *PRCC::NTRK1-*rearrangement.

### Statistical analyses

Statistical analyses were performed using IBM SPSS Statistics for Windows, Version 29.0. (Armonk, NY: IBM Corp. Released 2022). For positive predictive values (PPV) and negative predictive values (NPV), prevalence was calculated to be 1.1%, as in addition to the here identified *NTRK*- fusion positive cases, six already published *NTRK*-fusion positive sarcomas have been diagnosed in the same time period [6]. Interobserver agreement was quantified by calculation of the intraclass correlation coefficients (ICC) using a two-way random-effects model (2 out of 5 randomly assigned rater per case) and was interpreted as follows: < 0.50 poor, between 0.5 and 0.75 moderate, between 0.75 and 0.9 good, and > 0.90 excellent agreement [11]. Cohn’s kappa was calculated with the assumption of each score being one rater and interpreted according to Landis and Koch [12]. Alluvial Plot was generated using SankeyMATIC (Version 1.2.0, repository: https://github.com/nowthis/sankeymatic).

## Results

### Mesenchymal tumors with positive pan-Trk (clone EPR17341) IHC staining

**Fig. 2:**
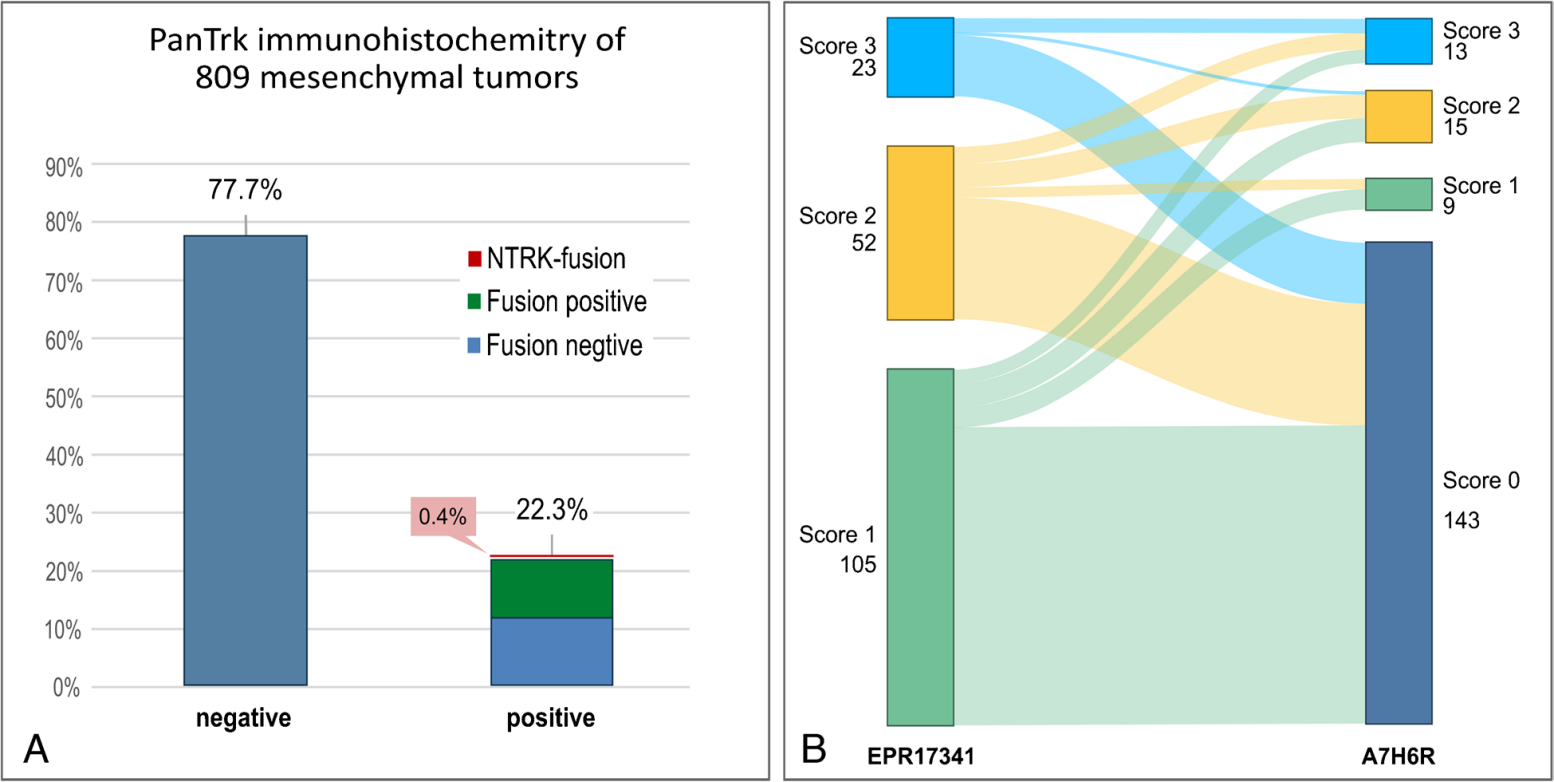
Pan-Trk immunohistochemistry. **A** Distribution of positive cases in relation to the underlying molecular alteration, clone EPR17341: fusion positive: fusion-driven entities (green); fusion negative: no rearrangement detected (blue) and *NKTR*-fusion: *NTRK* fusion positive cases (red). **B** Alluvial plot: staining intensities of all positive cases with clone EPR17341; *n* = 180, including *NTRK*-positive cases

Eight hundred nine (809)﻿ mesenchymal neoplasms were immunohistochemically analyzed using the pan-Trk clone EPR17341. 22.3% (180/809) of these tumors, stained positive (Table 1 and Fig. 2). Out of these 180 positively stained tumors, we confirmed and re-classified three (0.4%) tumors due to a retrospectively detected *NTRK1*-fusion (*TPR::NTRK1* (2x)*, LMNA::NTRK1*). In all three cases, a malignant peripheral nerve sheet tumor (MPNST) was initially favored (diagnoses made prior to 2013). In retrospect, none of these tumors harbored an immunohistochemical profile nor a *NF1* alteration that could have supported the diagnosis of a MPNST. Out of the 177 false positive stained tumors (21.9%), 21 cases demonstrated strong expression (score 3), 51 cases moderate expression (score 2), and 105 cases weak expression (score 1) (Table 1, Supplementary 1). Looking at the full cohort, this results in a specificity for the clone EPR17341 of 78%, sensitivity of 100%, PPV of 5%, and NPV of 100%. Inclusion of only the molecularly analyzed cases leads to a specificity of 68%, sensitivity of 100%, PPV of 3%, and NPV of 100%.

### High rate of false pan-Trk (clone EPR17341) positivity in fusion driven sarcomas

27.6% (223/809) of all included tumors, harbored a confirmed pathogenic gene fusion. 45.2% (80/177) of all false positive pan-Trk (EPR17341) cases accounted for such fusion driven sarcomas: DSRCT (100%, 2/2), high-grade endometrial stromal sarcoma (66%, 2/3), sarcoma with *BCOR* genetic alterations (57%, 4/7), extraskeletal myxoid chondrosarcoma (50%, 3/6), alveolar soft part sarcoma (50%, 1/2), dermatofibrosarcoma protuberans DFSP (43%, 6/14), Ewing sarcoma (39%,12/31), clear cell sarcoma of the soft tissue (37%, 3/8), myxoid liposarcomas (33% 20/60), synovial sarcoma (26%,17/64), solitary fibrous tumor SFT (21%, 7/33), low-grade endometrial stromal sarcoma (11%, 1/8), and low-grade fibromyxoid sarcoma (10%, 1/9). Additionally, positive pan-Trk (clone EPR17341) expression was detected in one intimal sarcoma harboring a *ZBTB16::ABL1*-fusion.

Interestingly, no false positivity was found in *CIC*-rearranged sarcomas (0/3), sclerosing epithelioid fibrosarcomas (0/4) and inflammatory myofibroblastic tumor (IMT) (0/3).

### Pan-Trk antibody clone-dependent staining sensitivity for mesenchymal tumors

All 180 positive cases for pan-Trk (EPR17341) were additionally stained with pan-Trk clone A7H6R. Strong positive expression was detected in all molecularly confirmed *NTRK1*-rearranged tumors (*n* = 3) and in 19.8% (34/177) of *NTRK wild type* cases. False positive staining with clone A7H6R was seen in schwannoma (*n* = 12), GIST (*n* = 7), UPS (*n* = 3), SFT (*n* = 3), DFSP (*n* = 2), Ewing sarcoma (*n* = 2), osteosarcoma (*n* = 2), clear cell sarcoma of the soft tissue (*n* = 1), EMC (*n* = 1), and MPNST (*n* = 1).

Of these 34 false positive cases, 11 tumors showed a strong expression (32%, score 3), 14 tumors a moderate expression (41%, score 2) and 9 tumors a weak expression (27%, score 1). The false positive staining intensity with clone A7H6R was stronger in 43% (15/34), the same in 46% (16/34) and weaker in 11% (4/34) of the cases compared to clone EPR1734. A comparison of distribution of staining intensities can be found in Fig. 2. Sarcomas with a *BCOR* genomic alterations (*n* = 7) and DSRCT (*n* = 2) did not stain false positive with pan-Trk clone A7H6R compared to clone EPR1734, 57% and 100%, respectively. In addition, 201 cases were selected and stained with the clone A7H6R, with an emphasis on fusion driven entities (*n* = 130 cases), as this group demonstrated a high rate of false positive cases, and 71 randomly selected, molecularly proven fusion negative cases. No false positive cases were detected in these additional cases (Table 1 and Fig. 2). Looking at all tested cases, these results indicate a specificity for the clone A7H6R of 91%, sensitivity of 100%, PPV of 19% and NPV of 100%. By only looking at the molecularly analyzed cases, the specificity is 94%, sensitivity 100%, PPV 16%, and NPV 100%.

### Interrater reliability across different clones

ICC for pan-Trk expression (EPR17341, scores 0–3) was excellent (*ĸ* = 0.978) (95% confidence interval (CI) 0.951–0.963). Distribution of the cases with no agreement between the first two pathologists (pan-Trk EPR17341, *n* = 36): 66% between score 0 and 1 (24 cases), 31% between score 1 and 2 (11 cases), and 3% between score 2 and 3 (1 case). Cohen’s kappa was almost perfect (*ĸ* = 0.884). ICC for pan-Trk expression (A7H6R) was excellent (*ĸ* = 0.974) (95% confidence interval (CI) 0.968–0.979). All cases with no agreement between the first two scoring pathologists (pan-Trk A7H6R, *n* = 5) were between score 0 and 1 (5 cases). Cohen’s kappa was almost perfect (*ĸ* = 0.915).

### False positive staining per compartment (cytoplasm, membranous, and nuclear)

False positive staining with clone ERP1734 was mainly seen in the cytoplasm 97% (173/177). The majority of these cases (60%) showed weak expression (score 1) followed by 29% with moderate expression (score 2) and 11% with strong expression (score 3). One clear cell sarcoma presented with a weak membranous expression and nuclear expression was seen in one MPNST (weak, score 1) as well as one intimal sarcoma with a strong expression harboring a *ZBTB16::ABL1* fusion (Fig. 2).

### NTRK fusion-positive spindle cell neoplasms

**Fig. 3:**
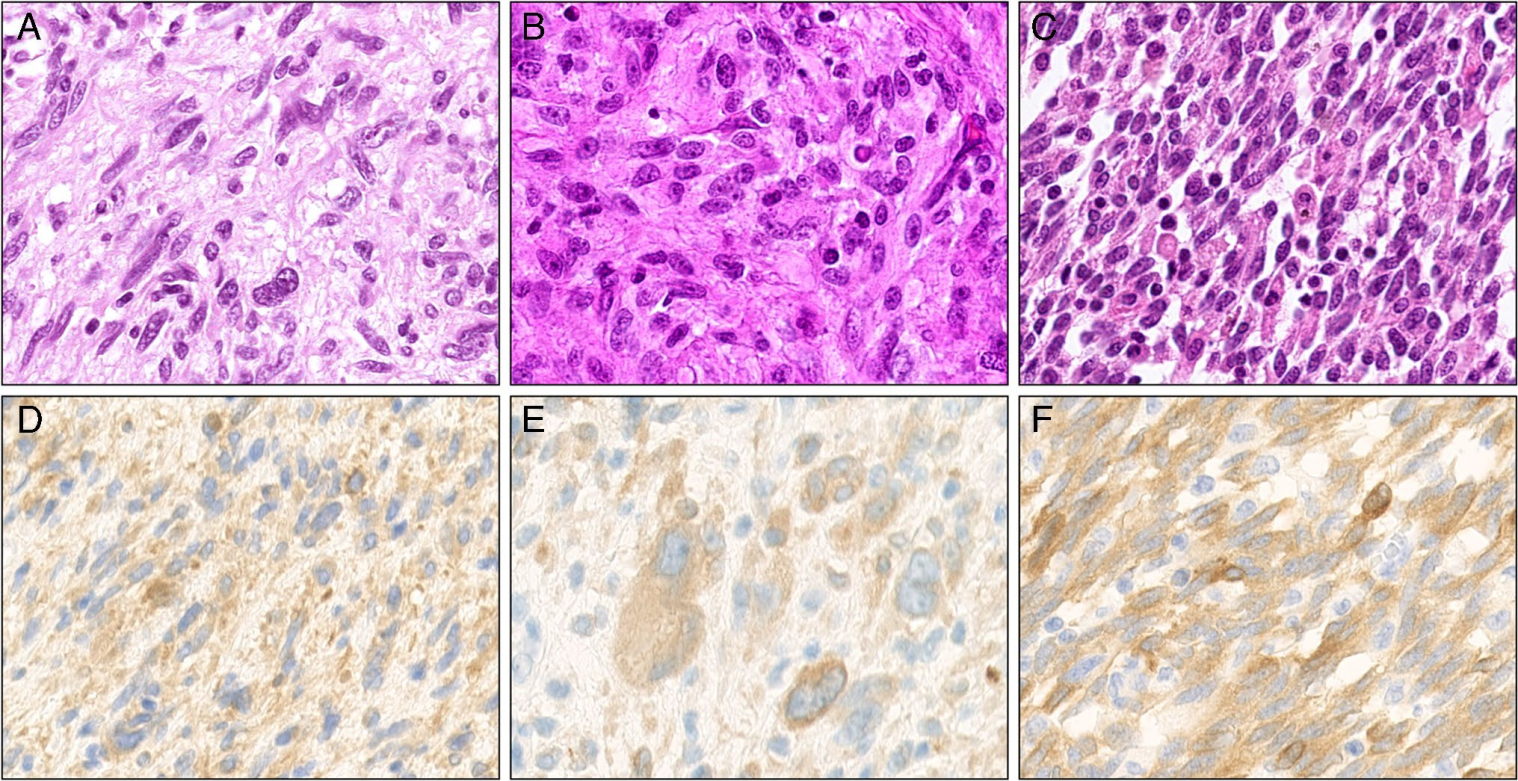
*NTRK* fusion positive cases: **A, D** *NTRK1::TPR* fusion; **B, E ***NTRK1::TPR* fusion; **C, F ***NTRK1::LMNA* fusion. Pan-Trk immunohistochemistry (A7H6R): **D** strong expression, **E** moderate expression, and **F** strong expression. Magnification 400x

A 3-year-old male patient presented with a 17-cm mass in the pancreas head. Total excision showed infiltration into the stomach, bowel and liver. The tumor was formed by a cellular population of spindled cells in a loose fashion (Fig. 3A). Immunohistochemistry showed a negative stain for EMA, desmin, myogenin, beta-catenin, CD21, CD23, und CD117. Pan-Trk expression showed strong cytoplasmic expression with both antibody clones, and S100 was also positive. Molecular analysis confirmed an *NTRK1::TPR* (chr1:156,844,212-chr1:186,329,420) fusion and a *CDKN2 A/B* deletion.

The second case was a 54-year-old male patient with a 9-cm mass at the lower left extremity. The tumor composed of loose spindle cells including some multinucleated, pleomorphic cells (Fig. 3E). Immunohistochemistry showed positive staining for pan-TRK (cytoplasm, with both antibody clones), S100, CD34, and weak positivity for SMA. Negative markers included PanCK AE1/AE3, desmin, ALK, and STAT6. Cytoplasmic pan-Trk expression was strong with clone EPR17341 and moderately with the clone A7H6R. Molecular analysis confirmed a *NTRK1::TPR* (chr1:156,845,347-chr1:186,337,016) fusion and a *CDKN2B* deletion.

The last case was an 8-year-old male patient with a soft tissue mass in the left foot, formed by a cellular population of spindled cells in short fascicles in a storiform way, which later metastasized to regional lymph nodes (Fig. 3C). Immunohistochemistry: S100 and CD34 were positive and pan-Trk showed a strong cytoplasmic positive stain with both antibody clones. Negative markers were SMA, desmin, PanCK AE1/AE3. Molecularly, a *NTRK::LMNA* (chr1:156,844,702-chr1:156,105,029) fusion was detected.

### PRCC:NTRK1-rearrangements

**Fig. 4:**
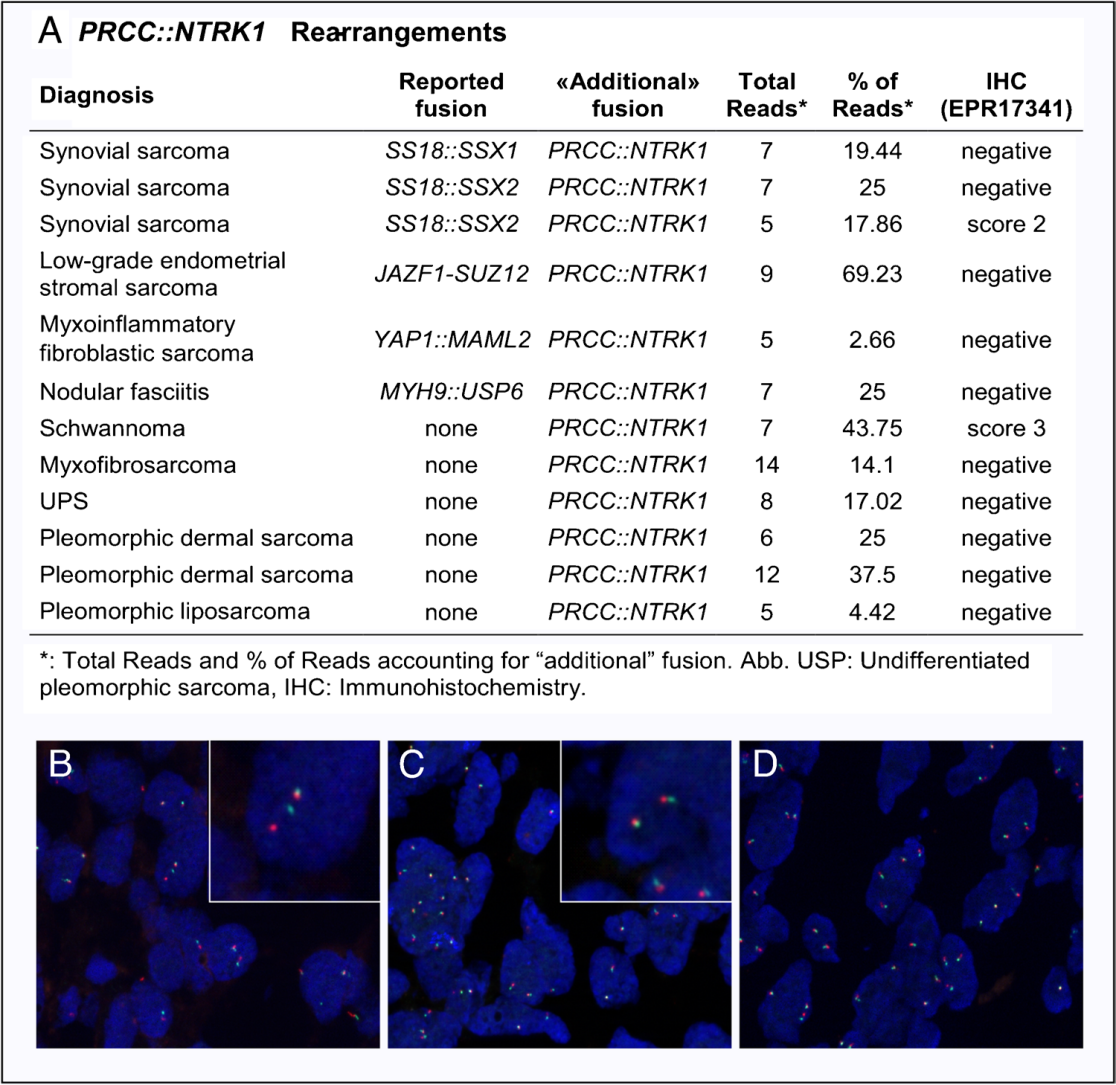
**A** Table with characteristics of *PRCC::NTRK1* fusion detected cases. **B** NTRK1 FISH Analysis. **B** NTRK fusion-positive case with split signals. **C** (Schwannoma) and **D** (UPS): normal (non-translocated) signals seen as two immediately adjacent red/green signals or as a fused yellow signal

During our routine molecular diagnostic work up (period 2022–2024), we found a non-pathogenic *PRCC::NTRK1-*rearrangement in 12 patients with mesenchymal neoplasms using a custom-made Archer™ FusionPlex™ Sarcoma Panel. Read counts were always low (< 15), and in 50% of the cases, an expected entity defining rearrangement was simultaneous detected, such as *S18::SSX* or *MYH9::USP6* (Fig. 4). The non-pathogenicity of this fusion was also confirmed by RNA bulk sequencing in one additional pediatric patient without a defining fusion. Immunohistochemistry for pan-Trk antibodies was negative in the vast majority of the cases (83%, 10/12). Additional *NTRK1*-FISH analysis, performed on these cases demonstrated normal (non-translocated) signals at 1q23.1 *NKTR1* region (Fig. 4).

## Discussion

The screening for a *NTRK1 - 3*-rearrangement by pan-Trk IHC is cheap, wildly available but not without challenges. In the literature, a lack of specificity and sensitivity has been recently discussed by multiple authors [5–7]. Positive pan-Trk staining with a cut-off of 1% should lead to RNA-based NGS reflex testing for the confirmation of the specific *NTRK1 - 3*-fusion [10]. Brčić I. et al. described a specificity problem for pan-Trk IHC in mesenchymal neoplasms, looking at 494 soft tissue sarcomas on a tissue micro array (TMA). They reported a false positive pan-Trk expression in 3.2% of the analyzed tumors [7]. In our study, we analyzed 809 mesenchymal tumors on whole slide section to appreciate intra-tumor heterogeneity and we report a specificity of 78% (177/809). Factors influencing such a difference might be different methods used and the different composition of entities.

Forty-five percent of the false positive stained cases harbored a non-*NTKR1 - 3*-gene fusion. False pan-Trk positivity has been previously described in various sarcomas harboring *BCOR*-genetic alterations as well as in DSRCT harboring *EWSR1::WT1* fusions by us and others. The false pan-Trk IHC positivity has been related to cross-reactions of the antibody in relation to upregulated gene expression of for example *NTRK3* that also plays an important role in tumorigenesis also in sarcomas [8, 13, 14]. A recent multicenter study using *NTRK3*-rearranged tumors showed that A7H6R might have a lower sensitivity than EPR17341 [15]. Negative A7H6R reactivity to BCOR sarcoma and DSRCT might suggests that this clone may not detect NTRK3 overexpression with sufficient sensitivity. However, the same study highlights the importance of a proper establishment of antibodies as the reference lab had identical results for A7H6R and EPR17341. In a previous study, we looked at antibody performance of three pan-Trk clones including A7H6R and EPR17341 [6]. On a smaller diverse subset of tumors, we had similar sensitivity of A7H6R and EPR17341. However, in the current study focusing on mesenchymal neoplasms only, 80% of the false positive cases using the clone EPR17341 were negative using the A7H6R clone.

Weak to strong positivity for pan-Trk immunohistochemistry was seen in 85% (31/37) of schwannomas using EPR17341 and twelve schwannomas using A7H6R (32%). This is most likely the fact because Schwann cells are derived from neural crest cells, where the family of transmembrane-receptor tyrosine kinases (TrkA, TrkB, and TrkC) plays an important role in neural development [16]. In this study, we exclusively included ancient schwannomas and schwannomas with regressive changes as they might cause a diagnostic challenge in biopsy specimens. An important differential diagnosis is MPNST, as several authors have described *NTRK*-rearrangements in the setting of *NF1* germline associated tumors. The relevance of these *NTRK-*fusions in the background of a *NF1* patients is not well understood, but individual patients might benefit from targeted therapy as described in the literature [17, 18].

It is of utmost relevance to identify patients that are benefitting from targeted therapy using TRK receptor tyrosine kinase inhibitors. Therefore, it is necessary to identify the tumors that harbor a pathogenic kinase fusion. As more institutions have access to molecular testing and also broader testing is performed, it also becomes very important, that only true pathogenic fusions are reported to not mislead any clinical management. We have recurrently detected a *PRCC::NTRK1-*rearrangement in our routine molecular practice using a RNA based custom-made Archer™ FusionPlex™ Sarcoma Panel. This fusion occurred with only low read counts and in few cases with simultaneous other known pathogenic fusions. Furthermore, negative immunohistochemistry with pan-Trk antibodies, negative *NKTR1*-FISH and bulk RNA sequencing in these cases, supports the lacking pathogenicity of this fusion. We rate this particular fusion a computational artifact in our custom-made Archer™ FusionPlex™ Sarcoma Panel. This gene fusion has so far once been reported in male breast cancer, based on whole genome sequencing [19]. The authors describe this fusion to be likely causing the activation of *NTRK1* but do not functionally validate it, which is important as Ameline et al. showed that *NTRK* fusions can also be non-functional and likely represent randomly occurring passenger alterations. We strongly believe that the detected fusion is not physiological relevant and should not be considered for targeted therapy. Proper understanding of the analytical tests used and validation of fusions detected on especially DNA but also on RNA level remains important. We and others [20] find pan-Trk IHC a very useful tool to help confirm a fusion as functional and pathological relevant.

The proposed *NTRK* testing algorithm in sarcomas includes factors such as tumor type and likelihood of harboring a *NTRK* fusion, as well as disease stage [9, 21, 22]. But the clinical and pathological features of *NTRK*-rearranged mesenchymal tumors are non-specific, making diagnosis challenging [23–27]. Screening by immunohistochemistry to detect increased levels of Trk protein has also been proposed, followed by molecular validation. From this study, we can conclude that the use of clone A7H6R for the screening of tumors with an *NTRK1 - 3*-rearrangement in mesenchymal neoplasms can lead to less false positive stains and eventually to less negative and costly reflex testing in mesenchymal tumors.

## Supplementary Information

Below is the link to the electronic supplementary material.

Supplementary file1 (XLSX 15 KB)

Supplementary file2 (XLSX 12 KB)

## Data Availability

The datasets used and/or analyzed during the current study are available from the corresponding author upon request.
